# Comparative Efficacy of Oral Chinese Patent Medicine for Chronic Prostatitis/Chronic Pelvic Pain Syndrome With Sexual Dysfunction: A Bayesian Network Meta-Analysis of Randomized Controlled Trials

**DOI:** 10.3389/fphar.2021.649470

**Published:** 2021-05-10

**Authors:** Yang Zhang, Hongzhao Ma, Tao Nan, Yongqiang Li, Wei Zheng, Zhihui Zhou, Xiaoyong Gong

**Affiliations:** ^1^ Shaanxi University of Chinese Medicine, Xianyang, China; ^2^ The Second Affiliated Hospital of Shaanxi University of Chinese Medicine, Xianyang, China

**Keywords:** network meta-analysis, chronic prostatitis/chronic pelvic pain syndrome, sexual dysfunction, oral medicine, Chinese patent medicine

## Abstract

**Background:** Oral Chinese patent medicine (OCPM) combined with western medicine (WM) are believed to be effective for the therapy of chronic prostatitis/chronic pelvic pain syndrome (CP/CPPS) with sexual dysfunction (SD). These western medicines mainly involve antibiotics, phosphodiesterase type-5 inhibitor (PDE-5i), α-blockers. But there is no randomized controlled trial (RCT) that directly compares the efficacy of different OCPM. Hence, we operated a network meta-analysis (NMA) to contrast the efficacy of different OCPM for CP/CPPS with SD.

**Methods:** Relevant studies were searched in PubMed, Cochrane Library, Web of Science, Embase, China National Knowledge Infrastructure (CNKI), Chinese Scientific Journal Database (VIP), and Wanfang database. All of the RCTs concentrated on the use of OCPM to cure CP/CPPS with SD from the inception of the databases to November 2020. We appraised the risk of bias under the Cochrane Handbook and CONSORT statement. The data were statistically analyzed *via* STATA 13.0 and WinBUGS 1.4.3 instrument.

**Results:** Altogether, 30 pieces of literature with 2,996 participants containing 11 oral Chinese patent medicine and 11 interventions were included in the NMA. In terms of The National Institutes of Health chronic prostatitis symptom index (NIH-CPSI), Qianlie Shutong Capsules (QLSTC) + WM had the most possible of being the optimal treatment. In the light of the International Index of Erectile Function (IIEF-5), Congrong Yishen Granules (CRYSG) + WM had the most possible of being the optimal treatment. Shugan Yiyang Capsules (SGYYC) + WM performed the highest likelihood efficacy under cluster rank graph combined NIH-CPSI and IIEF-5. Liuwei Dihuang Pills/Yougui capsules (LWDHP/YGC) + WM had highly possible to be the optimal treatment not only for the clinical effective rate of CP/CPPS but also for the clinical effective rate of SD. Considering four outcomes, QLSTC, CRYSG, SGYYC, LWDHP/YGC, Qianlie Beixi Capsules (QLBXC) plus WM were the best therapy approach for CP/CPPS with SD, especially LWDHP/YGC + WM and QLBXC + WM.

**Conclusion:** Based on the NMA, QLSTC, CRYSG, SGYYC, LWDHP/YGC, QLBXC plus WM demonstrated the maximum probability of being the optimal therapies. Owing to the limitations of this research, these results should be confirmed by elaborate RCTs.

**Systematic Review Registration:** [https://www.crd.york.ac.uk/prospero/], identifier [CRD42021224060].

## Introduction

“Prostatitis is classified as acute bacterial prostatitis (category I), chronic bacterial prostatitis (category II), chronic prostatitis (CP)/chronic pelvic pain syndrome (CPPS, category III, inflammatory IIIA, noninflammatory IIIB) and asymptomatic inflammatory prostatitis (category IV), according to the National Institutes of Health (NIH) classification system for prostatitis” (Krieger et al., 1999). CP/CPPS, a very common urinary system disease, with small and complex symptoms, which seriously affect patient’s quality of life. Furthermore, “men with CP/CPPS had a high probability to suffer sexual dysfunction (SD) than those without, such as erectile dysfunction (ED), premature ejaculation (PE), decreased sexual desire, ejaculatory pain and so on” (Li and Kang, 2016). “A meta-analysis involving 11,189 men showed that the prevalence of sexual dysfunction in men with CP/CPPS was high (SD was 62%, ED was 29% and PE was 40%), even though overall SD demonstrated a slightly decreasing trend, ED prevalence rate had an increasing trend in recent years” (Li and Chen, 2016). “A questionnaire survey was conducted among 1786 men indicated that the prevalence rate of SD in Chinese men with CP/CPPS is high (SD was 49%, ED was 15% and PE was 26%) and related to age” (Liang et al., 2004). Sexual dysfunction (SD) in our studies mainly refer to self-reported erectile dysfunction (ED) or premature ejaculation (PE), or both.

The studies on CP/CPPS with SD mainly concentrated on “epidemiology” (Liang et al., 2010; Hao et al., 2011), “risk factors” (Zhang et al., 2015a; Ma et al., 2020), and “relationship” (Chung et al., 2012), researches focus on therapeutics are scarce. “The National Institutes of Health (NIH) reached the consensus about prioritization of treatments for chronic prostatitis, rank 1 is antimicrobials (e.g., antibiotics), rank 2 is α-blockers (e.g., terazosin)” (Nickel et al., 1999). Lately, “α-blockers, antibiotics, and combinations of these therapies appear to achieve the greatest improvement in clinical symptom scores compared with placebo” (Anothaisintawee et al., 2011; Qin et al., 2016). However, the effectiveness of α-blockers and antibiotics has been controversial. Additionally, due to the long treatment time, we need to choose some drugs with fewer side effects and a longer course of treatment, so “Chinese patent medicine has become the best choice for us to treat chronic prostatitis with sexual dysfunction” (Zhong et al., 2013). “Oral Chinese patent medicine (OCPM) combined with western medicine (WM) has the beneficial effects for CP/CPPS with SD” (Wang et al., 2012; Feng et al., 2013).

**FIGURE 1:**
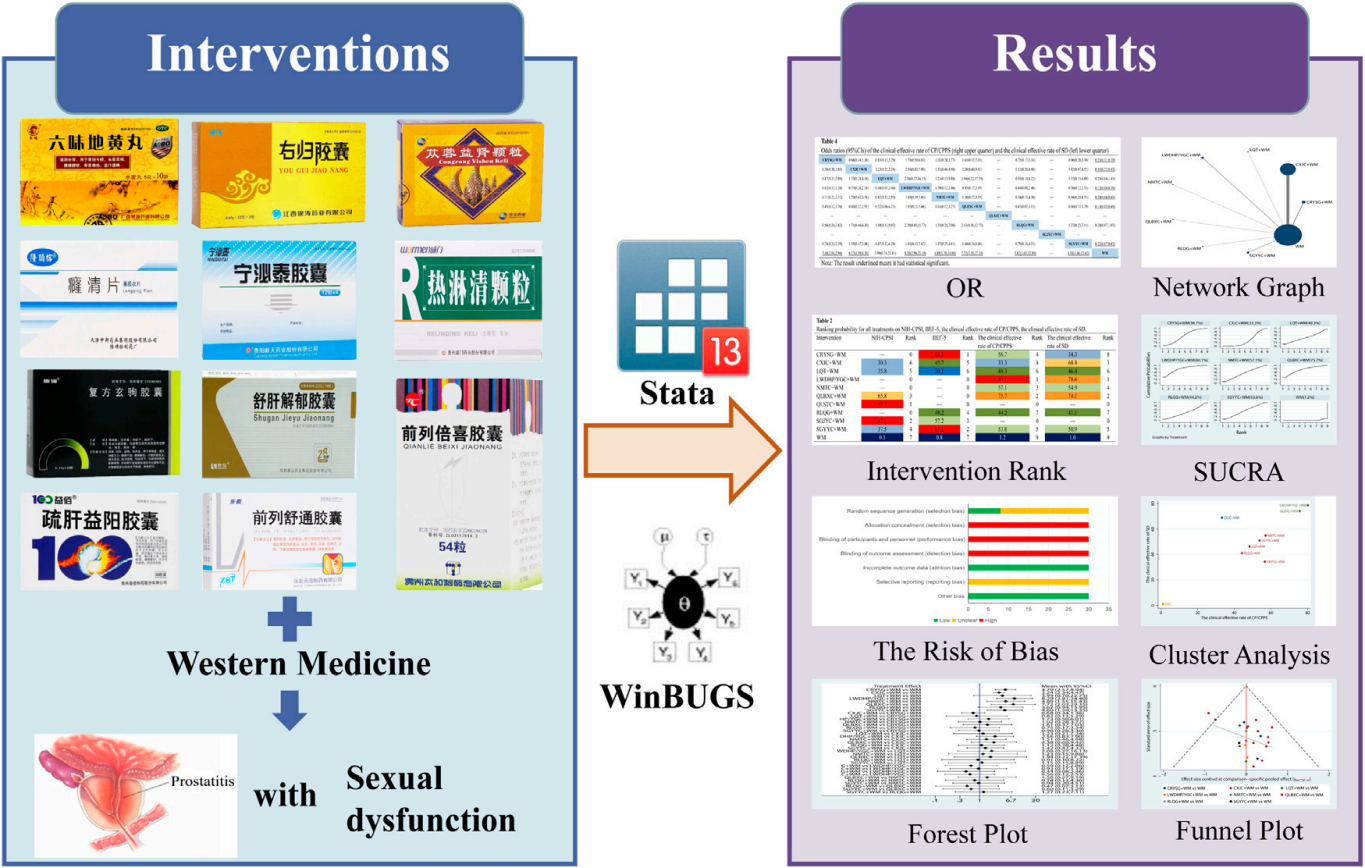
Graphic workflow for the NMA.

“Under traditional Chinese medicine (TCM) theories, CP means damp-heat and blood stasis syndromes” (Wang et al., 2016). Therefore, the top priority of treatment is to activate blood circulation and remove dampness. TCM combines syndrome differentiation with disease differentiation was more favorable to us to understand and treat CP. Syndrome differentiation is used to identify different types of a single disease to create a specific treatment plan. Hence, our research evaluated the clinical curative effect of OCPM combined with WM in patients who meet the standard therapy of CP/CPPS with SD: syntheses, individualization, and sequencing. Yet, there is no direct evidence showing the optimal OCPM for CP/CPPS with SD treatment. It is difficult to decide the superiority of treatment under a meta-analysis of pairwise comparisons. A network meta-analysis (NMA) combines existing evidence makes it possible to compare different treatment options at the same time. So, our research compared the curative effect of 11 OCPM combined with WM through NMA to reveal the optimal OCPM for CP/CPPS with SD and provide more perspectives for the choice of CP/CPPS with SD. The graphic workflow in our research is illustrated in Figure 1.

## Methods

### Eligibility Criteria

The RCTs that were published in English or Chinese were included if they met the following standard: 1) “Participants met criteria for CP/CPPS categories IIIA or IIIB according to the National Institutes of Health classification” (Krieger et al., 1999); 2) Participants met criteria for SD categories “erectile dysfunction (ED)” (Rosen et al., 1999) or “premature ejaculation (PE)” (Screponi et al., 2001), or both; 3) The interventions of experiment group involve OCPM add WM, the interventions of control group consist of WM alone or another OCPM plus WM. Besides, “these OCPMs must be included in the Pharmacopoeia of the People’s Republic of China”(Lin et al., 2020) or the data query system of the National Medical Products Administration website https://www.nmpa.gov.cn/; 4) The full text could be found and had sufficient data for collection, including the number of patients, age of patients, duration of therapy; 5) The study contained any of the following outcomes: NIH-CPSI scores, IIEF-5 scores, the clinical effectiveness of CP/CPPS, the clinical effectiveness of SD. The following formula was utilized: the clinical effectiveness (%) = (quantity of recovered patients + quantity of improved patients)/total quantity × 100%, The clinical effective rate of SD = the clinical effective rate of ED + the clinical effective rate of PE. The clinical effective rate of CP/CPPS was primary according to the reduction of the NIH-CPSI score, the clinical effective rate of ED was based on the increase of the IIEF-5 score and the clinical effective rate of PE was on the basis of the increase of Chinese Index of Premature Ejaculation (CIPE-5) score. These scores could be divided into three grades: recovery, improvement, and inefficiency. For response to treatment, various definitions were used in the original studies; recovery was determined when the decrements of the NIH-CPSI score >50%, recovery definition to reach the IIEF-5 score >21, recovery definition to reach the CIPE-5 score >17; improvement was defined when the decreases of the NIH-CPSI score >25%, improvement definition to reach the IIEF-5 score >15, improvement definition to meet 4-unit score increases in the CIPE-5 from baseline; the rest was inefficiency.

The following studies were excluded: studies involving patients who had 1) major psychological or somatic diseases; 2) the use of drugs that influence sexual function; 3) the use of antibiotics during the preceding 2 weeks; 4) missing or incorrect data; 5) experimental research, retrospective studies, conference abstracts, case reports, and reviews or meta-analyses.

### Data Sources and Retrieval Strategy

This research retrieves literature by employing the following databases from inception to November 2020: PubMed, Web of Science, Embase, Cochrane Library, CNKI, VIP, and Wanfang database. The free text words and medical subject headings (MeSH) were adopted. The restriction of language includes English and Chinese. Besides, we manually retrieved the reference of involved researches.

After overlaps were removed by EndNote X9, two researchers browsed the titles and abstracts of involved researches respectively. Besides, we also confirmed the potential articles. Any divergence could be solved through discussions or negotiations with a superior researcher.

### Data Extraction and Quality Evaluation

This information was extracted on the basis of the designed table: identity document (author’s name and year), participant’s characteristics (number of patients, age of patients, chronic prostatitis category, and sexual dysfunction category), methods of intervention, duration, outcomes, the random method.

Two authors evaluated the risk of bias. The intervention was systematically evaluated in randomized controlled trials and Cochrane Handbook. “The quality evaluation of the included RCTs focused on several key domains: selection bias (sequence generation and allocation concealment), performance bias (blinding of patients and personnel), detection bias (blinding of outcome assessors), attrition bias (incomplete outcome data), reporting bias (selective reporting), and other biases” (Higgins et al., 2011). Each of these options was evaluated as high, low or unclear. When described a valid generation of random number, blinding, and results, they will be categorized as low-risk, otherwise, they will be high-risk. Once described an ambiguous information, they will be categorized as an unclear risk. Any divergence could be solved through discussions or negotiations with a superior researcher.

### Statistical Analysis

The article adopted a Bayesian frame structure employing WinBUGS 1.4.3 and Stata 13.0 instrument to compute the outcomes. As for statistical processes, Random and fixed effects model was adopted for continuous and dichotomous variables, respectively. Simulation iterations were commanded to 200,000, adaptation iterations were required to10,000. Dichotomous variables or count data made use of odds ratios (ORs), continuous variables or measuring data chose mean difference (MD), as well as used their 95% confidence intervals (95% CIs). In addition, “the ranking probability of each treatment in different results was evaluated through the surface under the cumulative ranking area (SUCRA) value, a larger SUCRA value indicating a better treatment option” (Rücker and Schwarzer, 2015). Cluster analysis about variable results could be used to determine an optimal OCPM about therapy of CP/CPPS with SD under conducting a SUCRA. We didn’t implement the hypothesis of consistency because of non-close loops. Meanwhile, funnel graphs were plotted to identify the presence of publication bias. The forest graph, the risk of bias graph, and network plot were also illustrated.

## Results

### Study Characteristics of the Involved Researches

**FIGURE 2:**
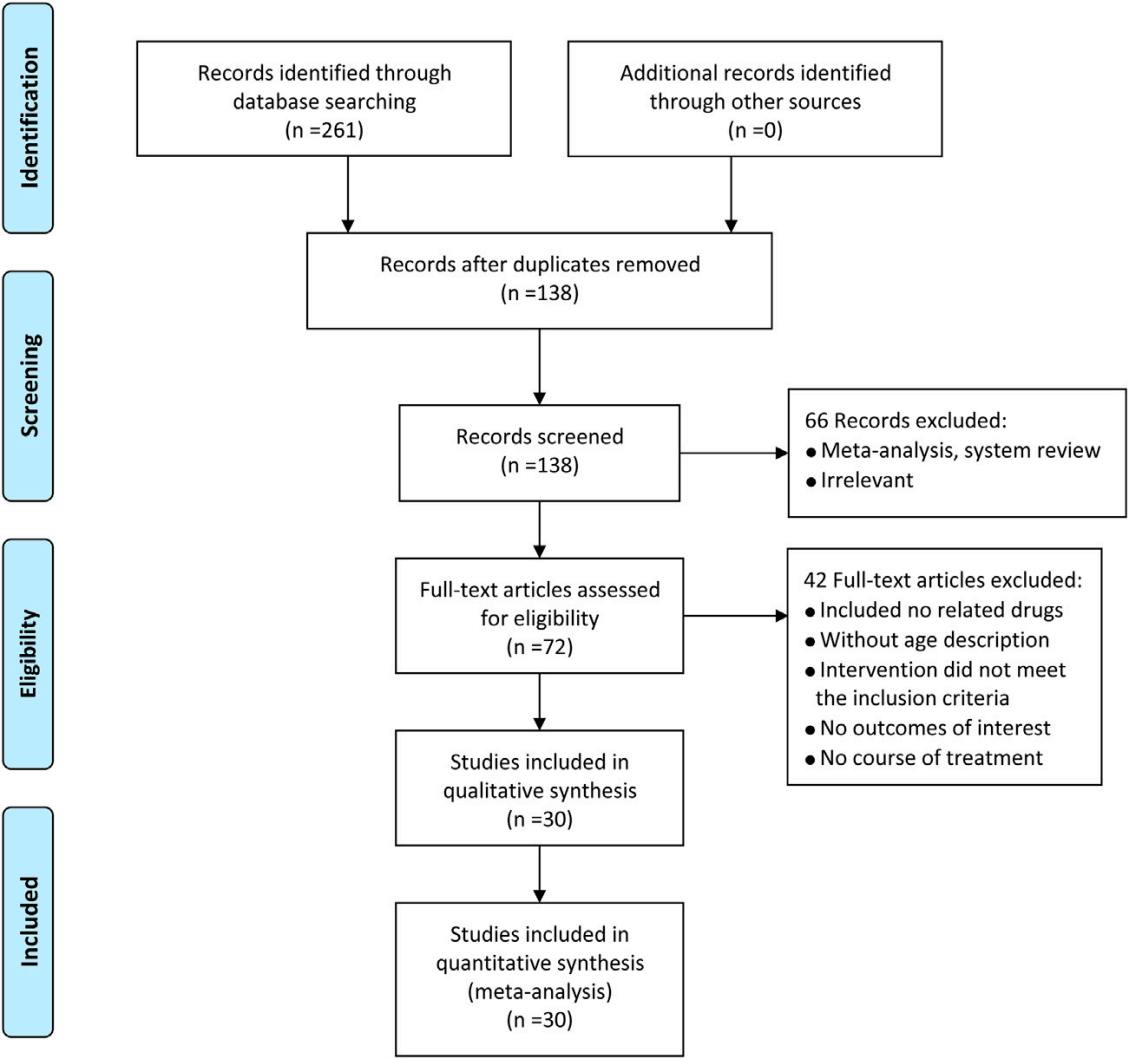
PRISMA flow diagram for eligible RCTs.

All of 261 pieces of literature from seven databases, 72 reserved by removing overlaps, reviews, unrelated researches, and research on animal. What's more, 42 articles were removed on account of the following causes: no related drugs, without age description, the intervention did not meet the inclusion conditions, no results of interest, no course of treatment. Ultimately, 30 (Liu et al., 2009; Sa et al., 2009; Li et al., 2011; Guo, 2012; Li et al., 2012; Luo, 2012; Wang et al., 2012; Yang, 2012; Feng et al., 2013; Zheng and Li, 2013; Zhong et al., 2013; Chen et al., 2014; Zhang et al., 2014a; Zhang et al., 2014b; Chen et al., 2015; Lang and Xia, 2015; Zhang et al., 2015b; Liu and Chen, 2016; Liu et al., 2016a; Liu et al., 2016b; Su et al., 2016; Wu et al., 2017; Liu et al., 2018; Zhou, 2018; Li et al., 2019; Liu et al., 2019; Hong and Liu, 2020; Wang, 2020; Xia et al., 2020; Zhao, 2020) two-arm randomized controlled trials presented in China from 2009 to 2020 were covered in our study. The PRISMA flow diagram was depicted in Figure 2.

**FIGURE 3:**
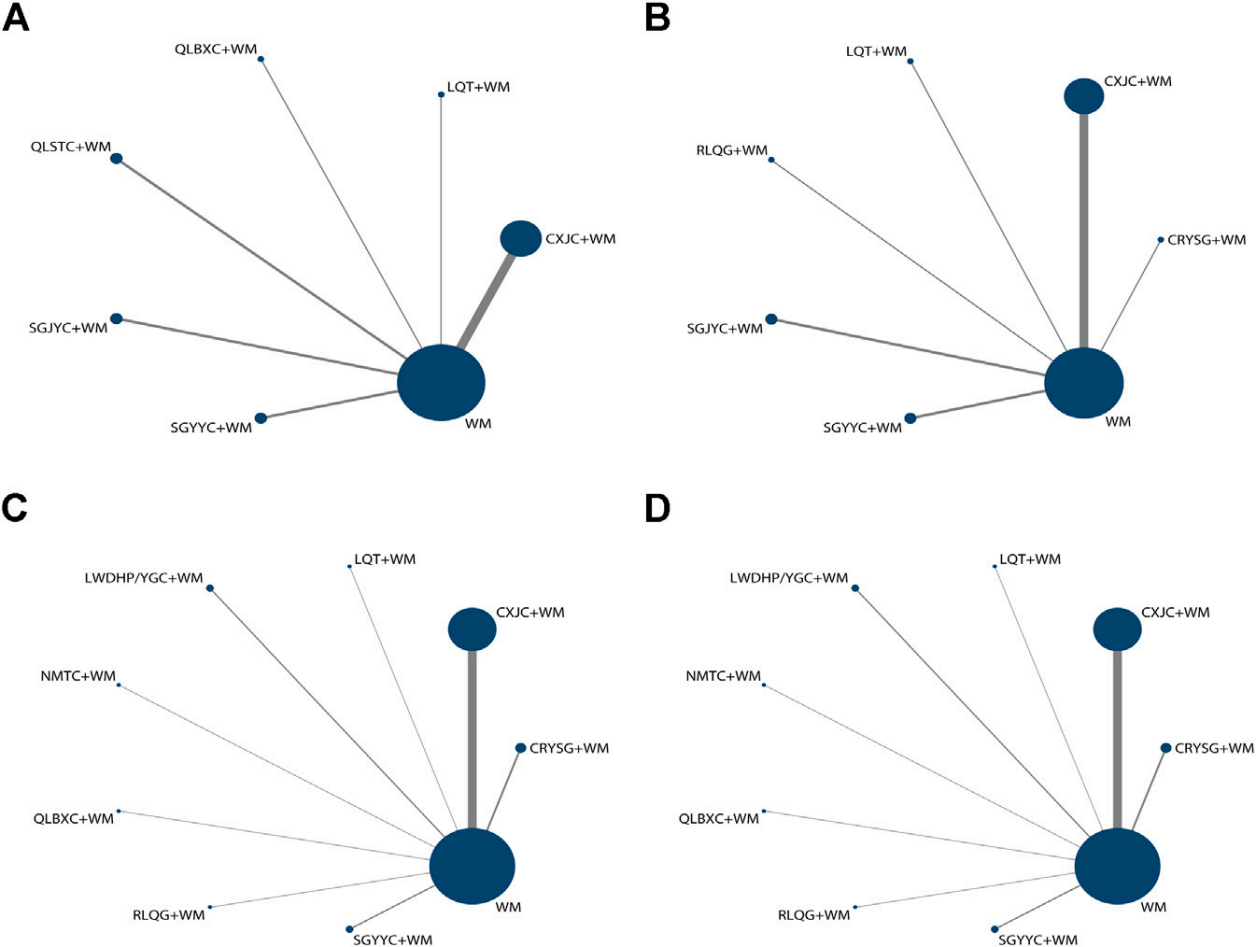
Network graphs for various outcomes **(A)** NIH-CPSI; **(B)** IIEF-5; **(C)** the clinical effective rate of CP/CPPS; **(D)** the clinical effective rate of SD.

Thirty studies with 2,996 patients (1,507 cases in the experimental group, 1,489 cases in the control group) involving 11 oral Chinese patent medicine and 11 interventions (LWDHP/YGC was one intervention based on syndrome differentiation, LWDHP for kidney-yin deficiency, YGC for kidney-yang deficiency) with CP/CPPS with SD (include PE = 5, ED = 10, SD = 15) were included in this NMA. The age range of the participants was approximately 15 to 66, and the young and middle-aged crowd made up the majority. Ten comparisons were appraised: Compound Xuanju Capsules+WM vs. WM (*n* = 15), Congrong Yishen Granules+WM vs. WM (*n* = 3), Liuwei Dihuang Pills/Yougui capsules+WM vs. WM (*n* = 2), Longqing Tablets+WM vs. WM (*n* = 1), Ningmitai Capsules+WM vs. WM (*n* = 1), Qianlie Beixi Capsules+WM vs. WM (*n* = 1), Qianlie Shutong Capsules+WM vs. WM (*n* = 2), Relinqing Granules+WM vs. WM (*n* = 1), Shugan Jieyu Capsules+WM vs. WM (*n* = 2) and Shugan Yiyang Capsules+WM vs. WM (*n* = 2). Western medicine regimen contained antibiotics, α-blockers, phosphodiesterase type-5 inhibitor (PDE-5i), an anti-inflammatory drug, pollen extract, etc. All the eligible Chinese patent medicine were taken orally, and the course of treatment ranged from 3 to 12 weeks, and most of the studies were 8 weeks. The detailed characteristics of included researches are demonstrated in Table 1, the patented formulations of involved literature are listed in Supplementary Table S1 and the network plots about diverse results could be found in Figure 3.

**TABLE 1:** Characteristics of included studies.

Study ID	Sample size/experiment/control	Age (Mean ± SD)		Prostatitis type	Sexualdysfunction type	Intervention		Duration(weeks)	Outcomes
		Experiment	Control			Experiment	Control		
Zhao (2020)	47/48	40.36 ± 1.41 (27∼66)	39.89 ± 1.47 (26∼66)	CP/CPPS	ED	QLSTC+WM	WM	12	①
Xia et al. (2020)	43/43	20～45	20～45	ⅢB	ED	LQT+WM	WM	8	①②③④
Wang (2020)	51/51	45.77 ± 5.45 (30∼60)	45.90 ± 5.23 (31∼60)	CP/CPPS	SD	RLQG+WM	WM	4	②③④⑦
Hong and Liu (2020)	28/28	33.1 ± 2.4 (17∼55)	32.1 ± 2.3 (18∼50)	IIIB	SD	SGJYC+WM	WM	8	①②⑥
Liu et al. (2019)	40/40	33.18 ± 4.56	32.84 ± 4.71	CP/CPPS	ED	CXJC+WM	WM	6	①②③④⑦
Li et al. (2019)	44/43	29.28 ± 4.07 (26∼38)	28.43 ± 4.43 (25∼38)	CP/CPPS	PE	QLSTC+WM	WM	3	①⑤
Liu et al. (2018)	50/50	22～51	21～49	CP/CPPS	ED	SGYYC+WM	WM	8	①②③④⑦
Zhou (2018)	49/49	34.57 ± 3.48 (24∼51)	34.82 ± 3.37 (23∼52)	CP/CPPS	SD	LWDHP/YGC+WM	WM	12	③④
Wu et al. (2017)	68/68	44.36 ± 5.22 (21∼64)	45.12 ± 5.04 (22∼66)	CP/CPPS	ED	CXJC+WM	WM	4	②
Su et al. (2016)	38/38	36.48 ± 5.21 (28∼54)	36.29 ± 5.34 (29∼52)	CP/CPPS	SD	CXJC+WM	WM	4	①②③④
Liu et al. (2016b)	43/43	23 ± 5 (18∼48)	23 ± 5 (18∼48)	IIIB	PE	QLBXC+WM	WM	4	①③④⑥
Liu and Chen, (2016)	52/48	28.6 ± 4.3 (22∼43)	28.6 ± 4.3 (22∼43)	CP/CPPS	SD	CRYSG+WM	WM	6	②③④
Liu et al., (2016a)	75/65	29 (19～41)	29 (19～41)	CP/CPPS	SD	CRYSG+WM	WM	8	④
Zhang et al. (2015b)	50/46	34.30 ± 8.15 (21～53)	34.30 ± 8.15 (21～53)	CP/CPPS	PE	CXJC+WM	WM	8	①③④⑥
Lang and Xia (2015)	49/49	32.4 ± 8.2 (21～52)	31.1 ± 8.9 (19～54)	IIIB	SD	SGJYC+WM	WM	8	①②⑥
Chen et al. (2015)	60/60	33.2 ± 8.7 (18～51)	32.2 ± 7.9 (19～54)	CP/CPPS	SD	CXJC+WM	WM	4	①②③④⑤⑦
Zhang et al. (2014b)	30/30	34.5 (20～56)	34.5 (20～56)	CP/CPPS	ED	CXJC+WM	WM	6	①②③④⑦
Zhang et al. (2014a)	93/105	30.2 (18～50)	30.2 (18～50)	CP/CPPS	ED	CXJC+WM	WM	8	①②③④⑦
Chen et al., 2014	45/45	35.32 ± 3.43 (26～45)	35.34 ± 3.41 27～45)	CP/CPPS	SD	CXJC+WM	WM	4	③④⑤
Feng et al. (2013)	40/40	25.5 ± 6.3 (16～41)	24.2 ± 5.9 (15～39)	CP/CPPS	ED	SGYYC+WM	WM	8	①②③④
Zeng ZF, 2013	40/40	23～47	23～47	CP/CPPS	SD	CXJC+WM	WM	4	③④
Zhong et al. (2013)	60/60	17.10 ± 5.16 (21～51)	(16.81 ± 5.26) (21～51)	CP/CPPS	SD	LWDHP/YGC+WM	WM	12	③④
Yang (2012)	49/49	32.4 (23～54)	31.8 (22～52)	CP/CPPS	PE	NMTC+WM	WM	8	③④⑦
Wang et al. (2012)	62/70	33.9 (19～58)	33.9 (19～58)	CP/CPPS	ED	CXJC+WM	WM	8	①②③④⑦
Luo (2012)	64/56	28 (20～43)	28 (20～43)	CP/CPPS	SD	CRYSG+WM	WM	8	③④
Li (2012)	50/40	35.2 (18～49)	35.2 (18～49)	CP/CPPS	SD	CXJC+WM	WM	6	③④⑤
Guo (2012)	20/20	36 (25～48)	34 (25～46)	CP/CPPS	SD	CXJC+WM	WM	4	③④
Li (2012)	31/31	31.5 (22～41)	31.5 (22～41)	CP/CPPS	PE	CXJC+WM	WM	4	③④
Sa (2009)	66/64	34.5 (19∼49)	34.5 (19～49)	CP/CPPS	ED	CXJC+WM	WM	6	③④
Liu (2009)	70/70	34.3 (20∼49)	34.3 (20～49)	CP/CPPS	SD	CXJC+WM	WM	6	③④⑤

①NIH-CPSI, ②IIEF-5, ③the clinical effective rate of CP/CPPS, ④the clinical effective rate of SD, ⑤SAS, ⑥CIPE-5, ⑦adverse reaction.

### Quality Assessment

**FIGURE 4:**
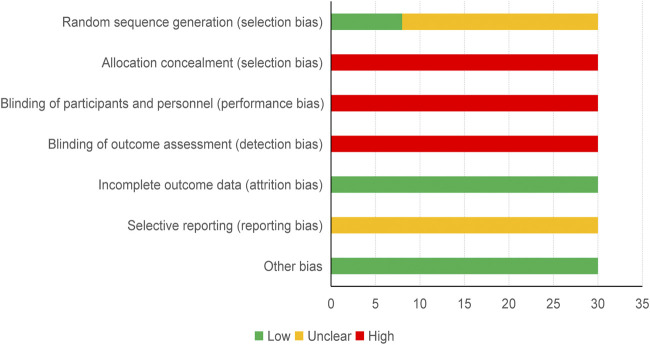
Risk of bias graph.

Two authors appraised the risk of bias in contained researches respectively by utilizing the Cochrane Risk of Bias Tool proposed by the Cochrane Handbook 5.1. Items assessed were as follows: Low-risk items: 1) seven studies about selective bias (explained utilized table of random numbers) and one study in selection bias (stated adopted lottery method). 2) All studies in attrition bias (reported outcome data completely). 3) All studies in other biases (stated a baseline of randomized controlled trials). High-risk events: 1) All studies about selection bias (did not adopt allocation concealment). 2) All studies in performance bias (did not utilize blinding of participants and personnel). 3) All researches in detection bias (did not report blinding of outcome assessment). Unclear risk items: 1) 22 studies in selection bias (did not state specific random method). 2) All studies in reporting bias (unclear selective reporting). A detailed description of the risk of bias evaluation is shown in Figure 4.

### Outcomes

#### NIH-CPSI Score

**FIGURE 5:**
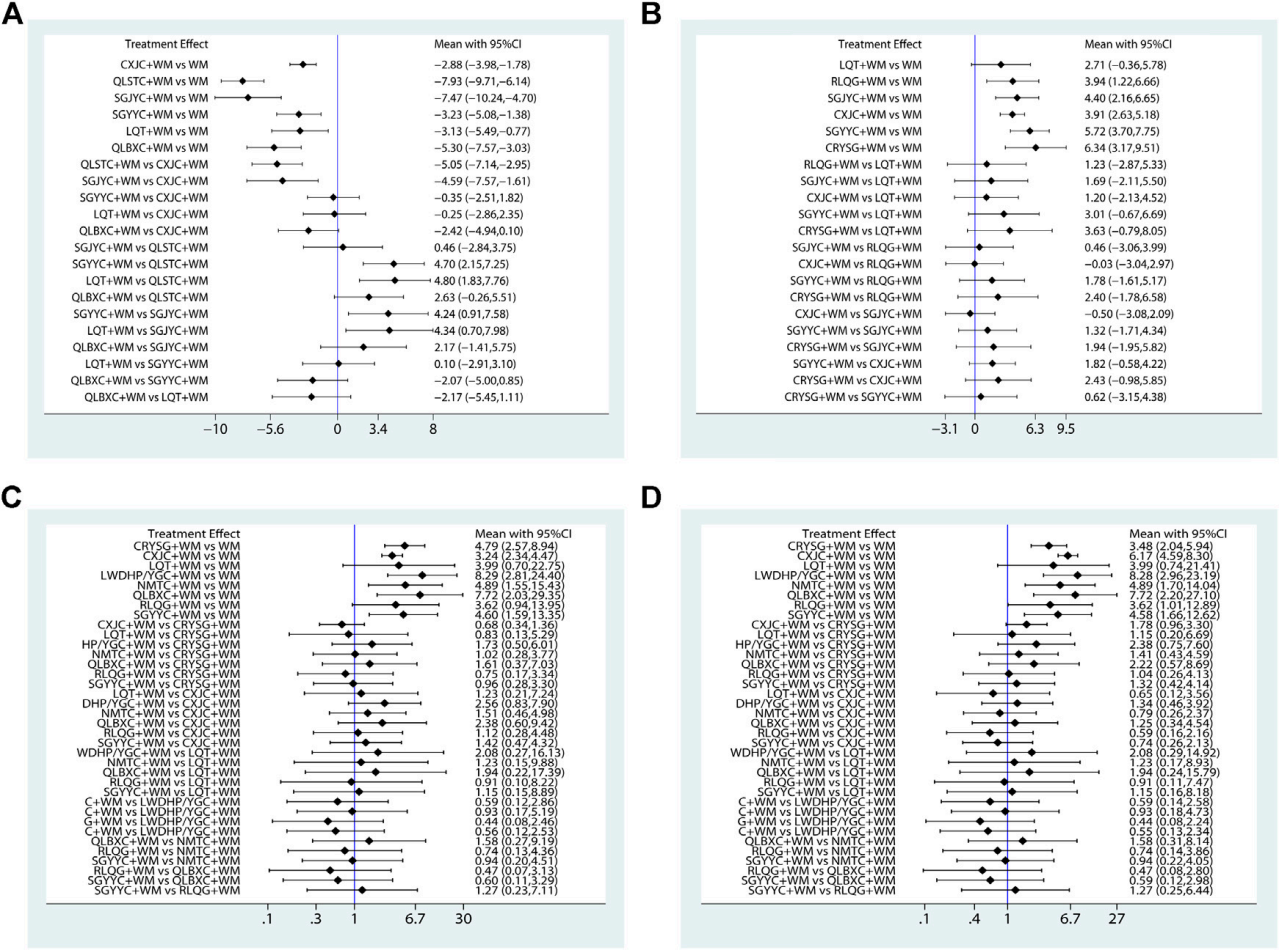
Forest graphs of Meta-analysis **(A)** NIH-CPSI; **(B)** IIEF-5; **(C)** the clinical effective rate of CP/CPPS; **(D)** the clinical effective rate of SD.

**FIGURE 6:**
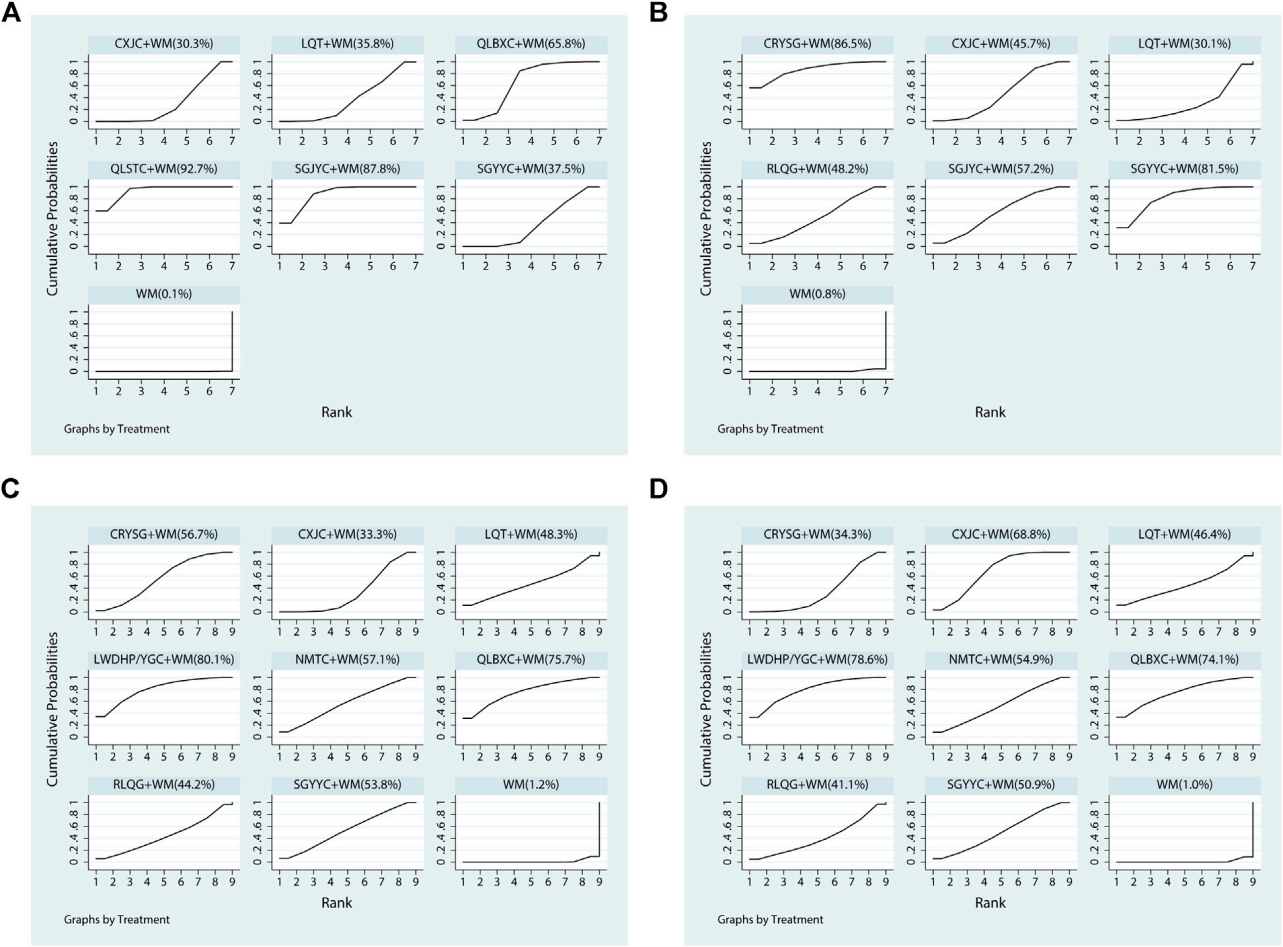
Plots of the surface under the cumulative ranking curves for all treatments **(A)** NIH-CPSI; **(B)** IIEF-5; **(C)** the clinical effective rate of CP/CPPS; **(D)** the clinical effective rate of SD.

All 15 pieces of research including six OCPM and seven interventions recorded the NIH-CPSI score. As the dominating results, the NIH-CPSI score was depicted in the right upper quarter of Table 2 and Figure 5A, CXJC+WM, LQT+WM, QLBXC+WM, QLSTC+WM, SGJYC+WM, and SGYYC+WM were better than the WM regimen alone in terms of the NIH-CPSI score. These results were statistically significant, MD and 95% CIs were 2.88 (1.78, 3.98), 3.13 (0.77, 5.49), 5.30 (3.03, 7.57), 7.93 (6.14, 9.71), 7.47 (4.70, 10.24) and 3.23 (1.38, 5.08), respectively. Additionally, QLSTC+WM was more effective than LQT+WM (MD = −4.80, 95% CI = −7.76 to −1.84) and CXJC+WM (MD = −5.05, 95% CI = −7.14 to −2.95; SGJYC+WM was more effective than LQT+WM (MD = −4.34, 95% CI = −7.98 to −0.70) and CXJC+WM (MD = −4.59, 95% CI = −7.57 to −1.61). According to the calculated probabilities of Figure 6A and Table 3, QLSTC+WM (92.7%) was the optimal combination in decreasing NIH-CPSI score, SGJYC+WM (87.8%) was number two, and QLBXC+WM (65.8%) was the third.

**TABLE 2:** Mean difference (95% CIs) of NIH-CPSI (right upper quarter) and IIEF-5 (left lower quarter).

CRYSG+WM	—	—	—	—	—	—	—	—	—	—
2.42 (−0.99, 5.84)	**CXJC+WM**	—	−0.25 (−2.86, 2.35)	—	−2.42 (−4.94, 0.10)	−5.05 (−7.14, −2.95)	—	−4.59 (−7.57, −1.61)	−0.35 (−2.51, 1.81)	2.88 (1.78, 3.98)
—	—	**LWDHP/YGC+WM**	—	—	—	—	—	—	—	—
3.62 (−0.79, 8.03)	1.20 (−2.13, 4.52)	—	**LQT+WM**	—	−2.17 (−5.44, 1.10)	−4.80 (−7.76, −1.84)	—	−4.34 (−7.98, −0.70)	−0.10 (−3.10, 2.90)	3.13 (0.77, 5.49)
—	—	—	—	**NMTC+WM**	—	—	—	—	—	—
—	—	—	—	—	**QLBXC+WM**	−2.63 (−5.51, 0.26)	—	−2.17 (−5.75, 1.41)	2.07 (−0.85, 5.00)	5.30 (3.03, 7.57)
—	—	—	—	—	—	**QLSTC+WM**	—	0.46 (−2.84, 3.75)	4.70 (2.15, 7.24)	7.93 (6.14, 9.71)
2.39 (−1.78, 6.56)	−0.04 (−3.04, 2.97)	—	−1.23 (−5.33, 2.87)	—	—	—	**RLQG+WM**	—	—	—
1.92 (−1.96, 5.81)	−0.50 (−3.08, 2.09)	—	−1.69 (−5.50, 2.11)	—	—	—	−0.46 (−3.99, 3.06)	**SGJYC+WM**	4.24 (0.91, 7.57)	7.47 (4.70, 10.24)
0.61 (−3.15, 4.36)	−1.82 (−4.21, 0.58)	—	−3.01 (−6.69, 0.66)	—	—	—	−1.78 (−5.17, 1.60)	−1.32 (−4.34, 1.71)	**SGYYC+WM**	3.23 (1.38, 5.08)
6.33 (3.16, 9.50)	3.91 (2.63, 5.18)	—	2.71 (−0.36, 5.78)	—	—	—	3.94 (1.22, 6.66)	4.40 (2.16, 6.65)	5.72 (3.70, 7.75)	**WM**

The result underlined meant it had statistical significant.

**TABLE 3:** Ranking probability for all treatments on NIH-CPSI, IIEF-5, the clinical effective rate of CP/CPPS, the clinical effective rate of SD.

Intervention	NIH-CPSI	Rank	IIEF-5	Rank	The clinical effective rate of CP/CPPS	Rank	The clinical effective rate of SD	Rank
CRYSG+WM	—	0	86.5	1	56.7	4	34.3	8
CXJC+WM	30.3	6	45.7	5	33.3	8	68.8	3
LQT+WM	35.8	5	30.1	6	48.3	6	46.4	6
LWDHP/YGC+WM	—	0	—	0	80.1	1	78.6	1
NMTC+WM	—	0	—	0	57.1	3	54.9	4
QLBXC+WM	65.8	3	—	0	75.7	2	74.1	2
QLSTC+WM	92.7	1	—	0	—	0	—	0
RLQG+WM	—	0	48.2	4	44.2	7	41.1	7
SGJYC+WM	87.8	2	57.2	3	—	0	—	0
SGYYC+WM	37.5	4	81.5	2	53.8	5	50.9	5
WM	0.1	7	0.8	7	1.2	9	1.0	9

### IIEF-5 Score

All 14 pieces of literature including six OCPM and seven interventions recorded the IIEF-5 score. As the other dominating outcomes, the IIEF-5 score was demonstrated in the left lower quarter of Table 2 and Figure 5B, CRYSG+WM, CXJC+WM, RLQG+WM, SGJYC+WM, and SGYYC+WM were better than the WM regimen by itself according to the IIEF-5 score. These results were statistically significant, MD and 95% CIs were 6.33 (3.16, 9.50), 3.91 (2.63, 5.18), 3.94 (1.22, 6.66), 4.40 (2.16, 6.65), and 5.72 (3.70, 7.75), respectively. No statistical significance was proved in the rest of the treatments. Relying on the ranking probabilities of Figure 6B and Table 3, CRYSG+WM (86.5%) was the best combination in increasing the IIEF-5 score, followed by SGYYC+WM (81.5%) and SGJYC+WM (57.2%).

### The Clinical Effective Rate of CP/CPPS

All 24 pieces of research including nine OCPM and nine interventions recorded the clinical effective rate of CP/CPPS. It was considered to the main outcomes, was displayed in the right upper quarter of Table 4 and Figure 5C, CRYSG+WM, CXJC+WM, LWDHP/YGC+WM, NMTC+WM, QLBXC+WM, and SGYYC+WM were better than the WM regimen alone according to the clinical effective rate of CP/CPPS. These results were statistically significant, ORs and 95% CIs were 0.21 (0.11, 0.39), 0.31 (0.22, 0.43), 0.12 (0.04, 0.36), 0.20 (0.06, 0.65), 0.13 (0.03, 0.49), and 0.22 (0.07, 0.63), respectively. Nevertheless, other interventions had no statistical difference. On the basis of the ranking probabilities of Figure 6C and Table 3, LWDHP/YGC+WM (80.1%) was the optimum combination in improving the clinical effective rate of CP/CPPS, QLBXC+WM (75.7%) was number two, and NMTC+WM (57.1%) was the third.

**TABLE 4:** Odds ratios (95% CIs) of the clinical effective rate of CP/CPPS (right upper quarter) and SD (left lower quarter).

CRYSG+WM	0.68 (0.34, 1.36)	0.83 (0.13, 5.29)	1.73 (0.50, 6.01)	1.02 (0.28, 3.77)	1.61 (0.37, 7.03)	—	0.75 (0.17, 3.34)	—	0.96 (0.28, 3.30)	0.21 (0.11, 0.39)
0.56 (0.30, 1.05)	**CXJC+WM**	1.23 (0.21, 7.24)	2.56 (0.83, 7.90)	1.51 (0.46, 4.98)	2.38 (0.60, 9.42)	—	1.12 (0.28, 4.48)	—	1.42 (0.47, 4.32)	0.31 (0.22, 0.43)
0.87 (0.15, 5.09)	1.55 (0.28, 8.54)	**LQT+WM**	2.08 (0.27, 16.13)	1.23 (0.15, 9.88)	1.94 (0.22, 17.39)	—	0.91 (0.10, 8.22)	—	1.15 (0.15, 8.89)	0.25 (0.04, 1.43)
0.42 (0.13, 1.34)	0.75 (0.26, 2.18)	0.48 (0.07, 3.46)	**LWDHP/YGC+WM**	0.59 (0.12, 2.86)	0.93 (0.17, 5.19)	—	0.44 (0.08, 2.46)	—	0.56 (0.12, 2.53)	0.12 (0.04, 0.36)
0.71 (0.22, 2.32)	1.26 (0.42, 3.78)	0.82 (0.11, 5.93)	1.69 (0.39, 7.40)	**NMTC+WM**	1.58 (0.27, 9.19)	—	0.74 (0.13, 4.36)	—	0.94 (0.20, 4.51)	0.20 (0.06, 0.65)
0.45 (0.12, 1.76)	0.80 (0.22, 2.91)	0.52 (0.06, 4.21)	1.07 (0.21, 5.44)	0.63 (0.12, 3.27)	**QLBXC+WM**	—	0.47 (0.07, 3.13)	—	0.60 (0.11, 3.29)	0.13 (0.03, 0.49)
—	—	—	—	—	—	**QLSTC+WM**	—	—	—	—
0.96 (0.24, 3.82)	1.71 (0.46, 6.30)	1.10 (0.13, 9.07)	2.29 (0.45, 11.77)	1.35 (0.26, 7.06)	2.13 (0.36, 12.75)	—	**RLQG+WM**	—	1.27 (0.23, 7.11)	0.28 (0.07, 1.07)
—	—	—	—	—	—	—	—	**SGJYC+WM**	—	—
0.76 (0.24, 2.39)	1.35 (0.47, 3.88)	0.87 (0.12, 6.20)	1.81 (0.43, 7.67)	1.07 (0.25, 4.61)	1.68 (0.34, 8.46)	—	0.79 (0.16, 4.01)	—	**SGYYC+WM**	0.22 (0.07, 0.63)
3.48 (2.04, 5.94)	6.17 (4.59, 8.30)	3.99 (0.74, 21.41)	8.28 (2.96, 23.19)	4.89 (1.70, 14.04)	7.72 (2.20, 27.10)	—	3.62 (1.01, 12.89)	—	4.58 (1.66, 12.62)	**WM**

The result underlined meant it had statistical significant.

### The Clinical Effective Rate of SD

All 24 pieces of literature including nine OCPM and nine interventions recorded the clinical effective rate of SD. As the other staple outcomes, the clinical effective rate of SD was depicted to the left lower part of Table 4 and Figure 5D, CRYSG+WM, CXJC+WM, LWDHP/YGC+WM, NMTC+WM, QLBXC+WM, RLQG+WM, and SGYYC+WM were better than the WM regimen alone accordance with the clinical effective rate of SD. These results were statistically significant, ORs and 95% CIs were 3.48 (2.04, 5.94), 6.17 (4.59, 8.30), 8.28 (2.96, 23.19), 4.89 (1.70, 14.04), 7.72 (2.20, 27.10), 3.62 (1.01, 12.89), and 4.58 (1.66, 12.62), respectively. No statistical significance was testified in the rest of the treatments. According to the ranking probabilities of Figure 6D and Table 3, LWDHP/YGC+WM (78.6%) was the first-rank combination in improving the clinical effective rate of SD, followed by QLBXC+WM (74.1%) and CXJC+WM (68.8%).

### SAS, CIPE-5, and Adverse Reaction

All five pieces of research including 2 OCPM and 3 interventions recorded the Self-rating anxiety scale (SAS), four studies involving three OCPM, and four interventions reported the Chinese Index of Premature Ejaculation (CIPE-5) and eight studies involving four OCPM and five interventions reported the adverse reaction. No statistical analysis was performed on these secondary outcomes on account of the scarcity of studies and inconsistent evaluation criteria.

### Cluster Analysis

**FIGURE 7:**
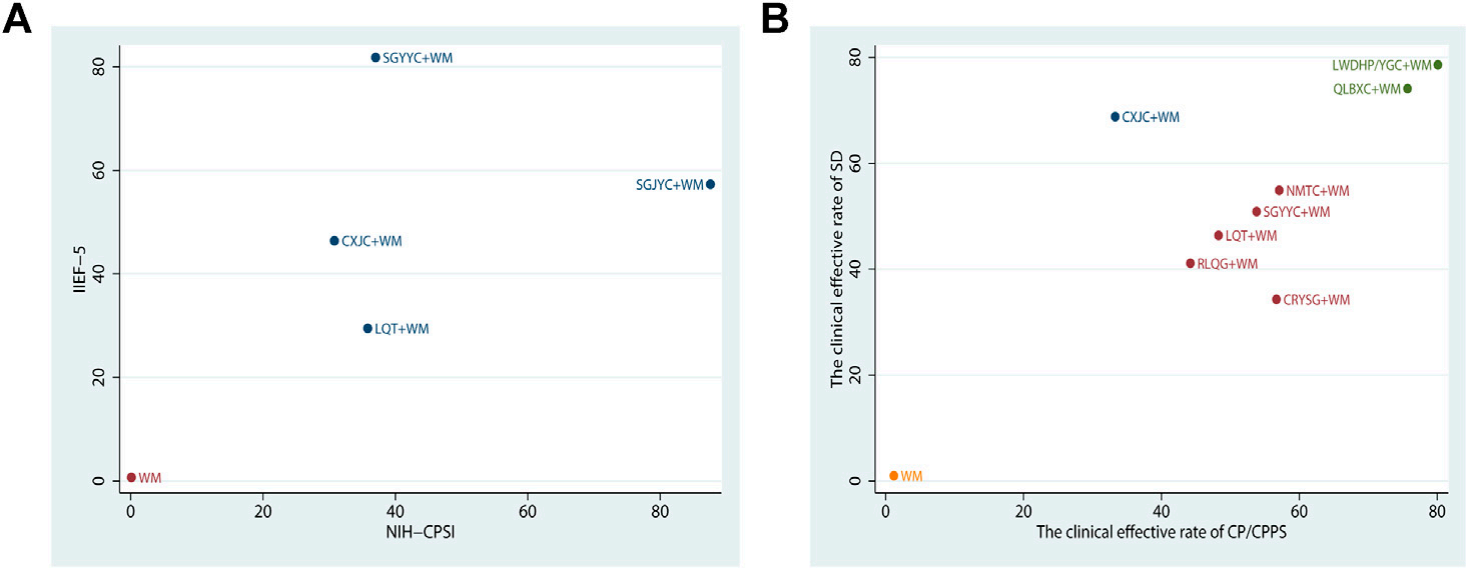
Cluster analysis plots. Interventions located in the upper right corner indicate optimal combination therapy for two different outcomes.

The influences of interventions in two diverse results were synthetically contrasted by cluster analysis. Two sets of cluster analyses were conducted in our study, containing NIH-CPSI and IIEF-5, the clinical effective rate of CP/CPPS and the clinical effective rate of SD, respectively. The results are shown in Figure 7. Through synthetical analysis by means of cluster analysis, SGJYC+WM had the better efficacy in decreasing the NIH-CPSI score, SGYYC+WM had the better curative effect in increasing the IIEF-5 score. Nevertheless, there was no optimum combination associated with a preferable response in both aspects. On the other group of cluster analysis, LWDHP/YGC+WM might possess the best efficacy in improving the clinical effective rate of CP/CPPS and SD, QLBXC+WM was in hot pursuit, their efficacy in the therapy of CP/CPPS with SD is worthy of attention.

### Publication Bias

**FIGURE 8:**
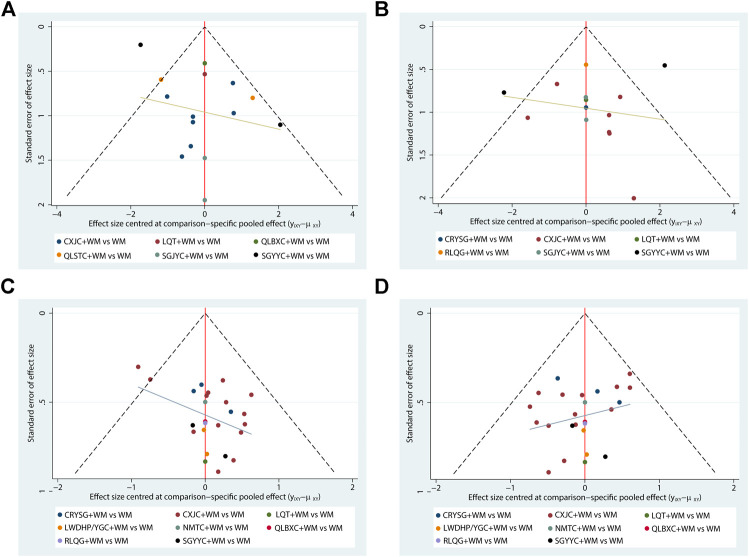
Funnel plots **(A)** NIH-CPSI; **(B)** IIEF-5; **(C)** the clinical effective rate of CP/CPPS; **(D)** the clinical effective rate of SD.

A funnel graph for four major outcomes was presented in Figure 8 to evaluate publication bias. All of the funnel graphs were not completely symmetrical visually, and each of the adjusted auxiliary lines was not perpendicular to the centerline. Thus, there may be significant publication bias.

## Discussion

### Summary of Main Findings

According to the information from 30 involved pieces of research with four primary outcomes, this article systematically appraised the efficacy of 11 commonly used OCPM (CRYSG, CXJC, LQT, LWDHP, NMTC, QLBXC, QLSTC, RLQG, SGJYC, SGYYC, and YGC) combined with WM in treating CP/CPPS with SD by taking advantage of network meta-analysis method. It’s worth noting that LWDHP/YGC belonged to one intervention through adopting dialectical treatment, LWDHP for kidney-yin deficiency, YGC for kidney-yang deficiency. On the account of the result of NMA, most OCPM combined with WM presented better results than utilizing WM by itself in various outcomes, and these results between groups were statistical differences. On the account of SUCRA results, QLSTC + WM had the maximum probability to be the best treatment in the NIH-CPSI. While in IIEF-5, CRYSG + WM intervention might be the best intervention. LWDHP/YGC had highly possible to be the optimal therapy in both the clinical effective rate of CP/CPPS and the clinical effective rate of SD. The clustered ranking according to NIH-CPSI compared with IIEF-5 showed there was no optimum combination better than any other combination. Fortunately, the clustered ranking based on the clinical effective rate of CP/CPPS compared with the clinical effective rate of SD showed LWDHP/YGC + WM had highly possible to be the best treatment on both sides. Considering four outcomes, QLSTC, CRYSG, SGYYC, LWDHP/YGC, Qianlie Beixi Capsules (QLBXC) plus WM were the optimum treatment regimens for CP/CPPS with SD, especially LWDHP/YGC + WM and QLBXC + WM. Therefore, the efficacy of LWDHP/YGC + WM and QLBXC + WM in the therapy of CP/CPPS with SD was worthy of attention, but clinicians should also select appropriate methods on the basis of specific situations of clinical patients.

### Research Significance and Importance

This article utilizes a network meta-analysis approach to appraise the curative effect of OCPM for treating CP/CPPS with SD for the first time. Meanwhile, our studies involved utilized treatment based on syndrome differentiation in Traditional Chinese Medicine (TCM) theories (e.g., LWDHP for kidney-yin deficiency, YGC for kidney-yang deficiency) and “obtained satisfactory curative effect” (Zhong et al., 2013; Zhou, 2018). In fact, “From the diagnosis of TCM, it included primarily type of syndrome, pathogen, position or name of a disease, such as wind-chill virus cold, wind-heat mycoplasma pneumonia” (Hou and Liu, 2013). Meanwhile, “Western medicine also developed a six-point clinical phenotyping of CP/CPPS to create a specific symptomatic treatment plan (UPOINT system), these clinical domains contained urinary symptoms, psychosocial dysfunction, organ-specific findings, infection, neurologic/systemic, and tenderness of muscles” (Shoskes and Nickel, 2013). “People have different opinions on whether to add SD into the UPOINT system” (Magri et al., 2010; Ersan et al., 2016). Basing on the above theory and method of integrated Chinese and Western medicine, we ranked the outcomes for the sake of offer evidence and recommendations for clinical medication. Furthermore, from the view of mechanisms, on the one hand, antibiotics could control infection and α-blockers could relaxes smooth muscles in areas such as the prostate and bladder and improves lower urinary tract symptoms and pain except anxious emotion, on the other hand, traditional Chinese medicine theory believes that anxiety is related to liver stagnation, OCPM could regulate liver stagnation and improves deficiency of kidney and spleen to relieves anxious emotion, improves erectile function and inhibit premature ejaculation ultimately. So as to say, OCPM and WM complement each other, physiotherapy and psychotherapy are as important as pharmacotherapy in the therapy of CP/CPPS with SD, “multi-disciplinary treatment (MDT) schema is expected to be preferred treatment in future” (Cheng and Zhang, 2018; Yan et al., 2019).

### Limitations

Nevertheless, this research also possessed limitations. In the first place, among 30 literature, only eight literature that utilized the correct method of a random number, none of them adopted allocation concealment, applied the blind method (blind to participants and personnel), and reported blinding of outcome evaluation. The quality of the involved researches might not be high, which reduced the persuasiveness of research results. In the second place, limited by application area of OCPM, all researches were reported in China and published in a Chinese journal, lacking the resource in other languages, which is not beneficial to the international promotion of study outcomes. In the third place, most RCTs studied on OCPM + WM vs. WM, lacking direct study on comparisons between diverse OCPM combined with WM. Finally, this research didn’t limit the phenotyping of CP/CPPS (include III = 26, IIIB = 4) and SD (include PE = 5, ED = 10, SD = 15). Therefore, we need to further analyze the efficacy of various OCPM on CP/CPPS with SD in the future.

### Prospects

Eventually, we propound the following recommendations: firstly, multicenter randomized double-blind tests should be implemented in rigorous in the light of regulations when conducting RCTs. The method of a random number, allocation concealment, and blind method performance are committed steps that need to be attentive in the future study program. Secondly, it is important to perform more clinical researches compared to the effect of different OCPM to make up for the absence of study in this domain. Finally, because of recurrent frequently of CP/CPPS with SD, a subsequent program should be carried out to supervise the prognosis of participants.

## Conclusion

By means of this NMA, OCPM combined with WM offered significant benefits compared to WM by itself in the therapy of chronic prostatitis/chronic pelvic pain syndrome (CP/CPPS) with sexual dysfunction (SD). What’s more, among the OCPM, LWDHP/YGC + WM and QLBXC + WM should be worthy of attention. But, on account of the limitations of this article, these conclusions were supposed to be certified *via* multicenter, high-quality, and larger-sample randomized controlled trials.

## Data Availability

The original contributions presented in the study are included in the article/Supplementary Material, further inquiries can be directed to the corresponding author.

## Glossary

CI: Credible interval
CIPE-5: Chinese Index of Premature Ejaculation
CNKI: China National Knowledge Infrastructure
CP/CPPS: Chronic prostatitis/chronic pelvic pain syndrome
CRYSG: Congrong Yishen Granules
CXJC: Compound Xuanju Capsules
ED: Erectile dysfunction
IIEF-5: The International Index of Erectile Function
LQT: Longqing Tablets
LWDHP/YGC: Liuwei Dihuang Pills/Yougui capsules
MD: Mean difference
NIH-CPSI: The National Institutes of Health chronic prostatitis symptom index
NMA: Network meta-analysis
NMTC: Ningmitai Capsules
OCPM: Oral Chinese patent medicine
ORs: Odds ratios
PDE-5i: phosphodiesterase type-5 inhibitor
PE: Premature ejaculation
QLBXC: Qianlie Beixi Capsules
QLSTC: Qianlie Shutong Capsules
RCT: Randomized controlled trial
RLQG: Relinqing Granules
SAS: Self-rating anxiety scale
SD: Sexual dysfunction
SGJYC: Shugan Jieyu Capsules
SGYYC: Shugan Yiyang Capsules
SUCRA: Surface under the cumulative ranking area
TCM: Traditional Chinese medicine
WM: Western medicine
