# Supplementary material for: Comparative Efficacy of Oral Chinese Patent Medicine for Chronic Prostatitis/Chronic Pelvic Pain Syndrome With Sexual Dysfunction: A Bayesian Network Meta-Analysis of Randomized Controlled Trials
Source: Front Pharmacol. 2021 May 10;12:649470. doi: 10.3389/fphar.2021.649470 (PMC8143435; doi:10.3389/fphar.2021.649470)
Supplement: Supplementary file 1 [file Table1.docx]

**Table S1 |** Detailed information on oral Chinese patent medicines.

| **Oral Chinese patent medicine**  **(Name of the formulation)** | **Name of the herbal drug** | **Scientific name of the plant and animal** | **Composition with Chinese pinyin** |
| --- | --- | --- | --- |
| Compound Xuanju capsule | SILKY ANT, EPIMEDII FOLIUM, LYCII FRUCTUS, CNIDII FRUCTUS | Polyrhachis vicina Roger, Epimedium brevicomu Maxim. or Epimedium sagittatum (Sieb. et Zucc.) Maxim. or Epimedium pubescens Maxim. or Epimedium koreanum Nakai, Lycium barbarum L., Cnidium monnieri (L.) Cuss. | Heimayi, Yinyanghuo, Gouqizi, Shechuangzi |
| Congrong Yishen granule | SCHISANDRAE CHINENSIS FRUCTUS, CISTANCHES HERBA, CUSCUTAE SEMEN, PORIA, PLANTAGINIS SEMEN, MORINDAE OFFICINALIS RADIX | Schisandra chinensis (Turcz.) Baill., Cistanche deserticola Y.C.Ma or Cistanche tubulosa (Schenk) Wight, Cuscuta australis R.Br. or Cuscuta chinensis Lam., Poria cocos (Schw.) Wolf, Plantago asiatica L. or Plantago depressa Willd., Morinda officinalis How | Wuweizi, Roucongrong, Tusizi, Fuling, Cheqianzi, Bajitian |
| Liuwei Dihuang pill | REHMANNIAE RADIX PRAEPARATA, CORNI FRUCTUS, MOUTAN CORTEX, DIOSCOREAE RHIZOMA, PORIA, ALISMATIS RHIZOMA | Rehmannia glutinosa Libosch., Cornus officinalis Sieb. et Zucc., Paeonia suffruticosa Andr., Dioscorea opposita Thunb., Poria cocos (Schw.) Wolf, Alisma orientale (Sam.) Juzep. or Alisma plantago-aquatica Linn. | Shudihuang, Jiuyurou, Mudanpi, Shanyao, Fuling, Zexie |
| Longqing tablet | ALISMATIS RHIZOMA, PLANTAGINIS SEMEN, PATRINIA, LONICERAE JAPONICAE FLOS, MOUTAN CORTEX, HEDYOTIS DIFFUSA, PAEONIAE RADIX RUBRA, AGRIMONIAE HERBA, COPTIDIS RHIZOMA, PHELLODENDRI CHINENSIS CORTEX | Alisma orientale (Sam.) Juzep. or Alisma plantago-aquatica Linn., Plantago asiatica L. or Plantago depressa Willd., Patrinia scabiosaefolia or Patrinia villosa, Lonicera japonica Thunb., Paeonia suffruticosa Andr., Hedyotis diffusa Willd, Paeonia lactiflora Pall. or Paeonia veitchii Lynch, Agrimonia pilosa Ledeb., Coptis chinensis Franch. or Coptis deltoidea C.Y.Cheng et Hsiao or Coptis teeta Wall., Phellodendron chinense Schneid. | Zexie, Cheqianzi, Baijiangcao, Jinyinhua, Mudanpi, Baihuasheshecao, Chishao, Xianhecao, Huanglian, Huangbo |
| Ningmitai capsule | POLYGONUM CAPITATUM, IMPERATAE RHIZOMA, PIPERIS KADSURAE CAULIS, BERBERIDIS RADIX, AGRIMONIAE HERBA, HIBISCI MUTABILIS FOLIUM, FORSYTHIAE FRUCTUS | Polygonum capitatum Buch., Imperata cylindrica Beauv.var.major (Nees) C.E.Hubb., Piper kadsura (Choisy) Ohwi, Berberis soulieana Schneid. or Berberis wilsonae Hemsl. or Berberis poiretii Schneid. or Berberis vernae Schneid., Agrimonia pilosa Ledeb., Hibiscus mutabilis L., Forsythia suspensa (Thunb.) Vahl | Sijihong, Baimaogen, Dafengteng, Sankezhen, Xianhecao, Mufurongye, Lianqiao |
| Qianlie Beixi capsule | ADIANTUM CAPILLUS-VENERIS, GRYLLOTALPA, VACCARIAE SEMEN, GLEDITSIAE SPINA, ERINACEUS EUROPAEUS | Adiantum capillus-veneris L., Gryllotalpa unispina Saussure or Gryllotalpa orientalis Burmeister, Vaccaria segetalis (Neck.) Garcke, Gleditsia sinensis Lam., Erinaceus europaeus L. or Hemichianus dauricus Sundevall. or Hemichianus auritus Gmelin. | Zhuzongcao, Lougu, Wangbuliuxing, Zaojiaoci, Ciweipi |
| Qianlie Shutong capsule | PHELLODENDRI CHINENSIS CORTEX, PAEONIAE RADIX RUBRA, ANGELICAE SINENSIS RADIX, CHUANXIONG RHIZOMA, SMILACIS GLABRAE RHIZOMA, SPARGANII RHIZOMA, ALISMATIS RHIZOMA, PORTULACAE HERBA, VERBENAE HERBA, SAXIFRAGA STOLONIFERA, BUPLEURI RADIX, CYATHULAE RADIX, GLYCYRRHIZAE RADIX ET RHIZOMA | Phellodendron chinense Schneid., Paeonia lactiflora Pall. or Paeonia veitchii Lynch, Angelica sinensis (Oliv.) Diels, Ligusticum chuanxiong Hort., Smilax glabra Roxb., Sparganium stoloniferum Buch. -Ham., Alisma orientale (Sam.) Juzep. or Alisma plantago-aquatica Linn., Portulaca oleracea L., Verbena officinalis L., Saxifraga stolonifera Curt., Bupleurum chinense DC. or Bupleurum scorzonerifolium Willd., Cyathula officinalis Kuan, Glycyrrhiza uralensis Fisch. or Glycyrrhiza inflata Bat. or Glycyrrhiza glabra L. | Huangbo, Chishao, Danggui, Chuanxiong, Tufuling, Sanleng, Zexie, Machixian, Mabiancao, Huercao, Chaihu, Chuanniuxi, Gancao |
| Relinqing granule | POLYGONUM CAPITATUM | Polygonum capitatum Buch. | Touhualiao |
| Shugan Jieyu capsule | HYPERICI PERFORATI HERBA, ACANTHOPANACIS SENTICOSI RADIX ET RHIZOMA SEU CAULIS | Hypericum perforatum L, Acanthopanax senticosus (Rupr.etMaxim.) Harms | Guanyejinsitao, Ciwujia |
| Shugan Yiyang capsule | TRIBULI FRUCTUS, BUPLEURI RADIX, VESPAE　NIDUS, PHERETIMA, HIRUDO, ASPONGOPUS, SPONGILLA, CNIDII FRUCTUS, POLYGALAE RADIX, CISTANCHES HERBA, CUSCUTAE SEMEN, SCHISANDRAE CHINENSIS FRUCTUS, MORINDAE OFFICINALIS RADIX, SCOLOPENDRA, ACORI TATARINOWII RHIZOMA | Tribulus terrestris L., Bupleurum chinense DC. or Bupleurum scorzonerifolium Willd., Polistes　olivaceous (DeGeer) or Polistes　japonicas　Saussure or Parapolybia　varia　Fabricius, Pheretima aspergillum (E.Perrier) or Pheretima vulgaris Chen or Pheretima guillelmi (Michaelsen) or Pheretima pectinifera Michaelsen, Whitmania pigra Whitman or Hirudo nipponica Whitman or Whitmania acranulata Whitman, Aspongopus chinensis Dallas, Spongilla fragills (Leidy), Cnidium monnieri (L.) Cuss., Polygala tenuifolia Willd. or Polygala sibirica L., Cistanche deserticola Y.C.Ma or Cistanche tubulosa (Schenk) Wight, Cuscuta australis R.Br. or Cuscuta chinensis Lam., Schisandra chinensis (Turcz.) Baill., Morinda officinalis How, Scolopendra subspinipes mutilans L. Koch, Acorus tatarinowii Schott | Jili, Chaihu, Fengfang, Dilong, Shuizhi, Jiuxiangchong, Zishaohua, Shechuangzi, Yuanzhi, Roucongrong, Tusizi, Wuweizi, Bajitian, Wugong, Shichangpu |
| Yougui capsule | REHMANNIAE RADIX PRAEPARATA, ACONITI LATERALIS RADIX PRAEPARATA, CINNAMOMI CORTEX, DIOSCOREAE RHIZOMA, CORNI FRUCTUS, CUSCUTAE SEMEN, CERVI CORNUS COLLA, LYCII FRUCTUS, ANGELICAE SINENSIS RADIX, EUCOMMIAE CORTEX | Rehmannia glutinosa Libosch., Aconitum carmichaelii Debx., Cinnamomum cassia Presl, Dioscorea opposita Thunb., Cornus officinalis Sieb. et Zucc., Cuscuta australis R.Br. or Cuscuta chinensis Lam., Cervus elaphus Linnaeus or Cervus Nippon Temminck, Lycium barbarum L., Angelica sinensis (Oliv.) Diels, Eucommia ulmoides Oliv. | Shudihaung, Fuzi, Rougui, Shanyao, Shanzhuyu, Tusizi, Lujiaojiao, Gouqizi, Danggui, Duzhong |
